# Annexin A3 Regulates Early Blood Vessel Formation

**DOI:** 10.1371/journal.pone.0132580

**Published:** 2015-07-16

**Authors:** Stryder M. Meadows, Ondine Cleaver

**Affiliations:** 1 Department of Cell and Molecular Biology, Tulane University, 2000 Percival Stern Hall, 6400 Freret St., New Orleans, LA, United States of America; 2 Department of Molecular Biology, University of Texas Southwestern Medical Center, 5323 Harry Hines Blvd., Dallas, Texas, United States of America; Katholieke Universiteit Leuven, BELGIUM

## Abstract

Annexins are a large family of calcium binding proteins that associate with cell membrane phospholipids and are involved in various cellular processes including endocytosis, exocytosis and membrane-cytoskeletal organization. Despite studies on numerous Annexin proteins, the function of Annexin A3 (Anxa3) is largely unknown. Our studies identify Anxa3 as a unique marker of the endothelial and myeloid cell lineages of *Xenopus laevis* during development. Anxa3 transcripts are also detected in endothelial cells (ECs) of zebrafish and mouse embryos, suggesting an important evolutionary function during formation of blood vessels. Indeed, Anxa3 loss-of-function experiments in frog embryos reveal its critical role during the morphogenesis of early blood vessels, as angioblasts in MO injected embryos fail to form vascular cords. Furthermore, *in vitro* experiments in mammalian cells identify a role for Anxa3 in EC migration. Our results are the first to reveal an *in vivo* function for Anxa3 during vascular development and represent a previously unexplored aspect of annexin biology.

## Introduction

Annexins are a class of Ca^2+^ binding proteins present in evolutionarily distant species ranging from simple protists to humans [1]. Annexins are characterized by an “Annexin” core, which binds to negatively charged phospholipids of the inner cell membrane in a Ca^2+^ dependent manner. The bulk of the annexin open reading frame encodes the annexin core, which is comprised of four annexin repeat domains approximately 70 amino acid residues each (Anxa6 contains six annexin domains). In addition, each family member has a short, unique N-terminal domain thought to form complexes with cytosolic proteins.

Annexins have a broad range of functions, but are thought to effectively link calcium signaling and membrane dynamics. Annexin roles have been identified in endocytosis, exocytosis, membrane organization and migration through association of partner proteins, including members of the S100 family of calcium-binding EF-hand proteins [2–12].

Currently, 12 Annexin genes have been identified in mammals, however only a few have been shown to have roles in endothelial cell (EC) biology. Anxa1, 2, 5 and 6 are expressed in ECs of humans, mice and chickens [13–16], (GEISHA-Gallus Expression In Situ Hybridization Analysis). In regards to vascular biology, Anxa2 has garnered the most attention, with various roles reported *in vivo* and *in vitro*. Depletion of Anxa2 in mice is reported to result in defective fibrinolysis and neoangiogenesis [15,17,18], while in cultured ECs, Anxa2 is required for secretion of the von Willebrand factor (vWF) [4] and junctional integrity [19].

At present, the function of Anxa3, if any, during blood vessel development is not understood. Park et al. [20] showed that Anxa3 can induce cell migration and plexus formation in cultured ECs, possibly through increased production of vascular endothelial growth factor (VEGF) via transactivation of the hypoxia-inducible factor-1 (HIF1) pathway. However since then, progress in our understanding of Anxa3 function in ECs has been limited. Here, we further explore the role of Anxa3 during blood vessel formation, using *in vitro* and *in vivo* systems. We find that Anxa3 is expressed in the endothelial lineages of fish, frog and mice. Utilizing morpholino-based knockdown strategies in *Xenopus laevis* embryos, we show that Anxa3 is required during vasculogenesis, the initial stage of blood vessel formation. Depletion of Anxa3 in frogs results in inhibition of angioblast coalescence and blood vessel morphogenesis, while loss of Anxa3 in cultured human ECs inhibits migratory behavior. In addition, we find that Anxa3 is one of the dominant Annexin family members expressed in the embryonic endothelium of frog and fish, whereas multiple Annexins are present in mammalian ECs, signifying an evolutionary distinction in overlapping functions between various species. This investigation further implicates annexin function in vascular biology and is the first to uncover a role for Anxa3 during vascular development.

## Materials and Methods

### Embryological microinjections and Morpholino Oligomers

Embryos were staged according to Nieuwkoop and Faber, 1994 [21]. Antisense morpholinos oligomers (MO) were stored in 50 mM HEPES buffer, pH 8.0 and heated for 10 minutes at 65° prior to use. MOs (25 and 50 ng) and mRNAs (500 pg and 1ng) were injected into 1 cell of 2-cell staged embryos in 0.4X MMR containing 6% Ficoll and cultured thereafter in 0.2X MMR. For Anxa3 MO specificity experiments using an mRNA tester construct consisting of the Anxa3 5’UTR plus the first 42 nucleotides of coding region fused to the coding sequence of green fluorescent protein (GFP), 1 cell staged embryos were injected and observed at approximately stage 12. Anxa3 MO1 was designed to the Anxa3 5’UTR (5’-GCAGTATATAGCGTTTCTAGTGGGA-3’) and Anxa3 MO2 was designed against Anxa3 5’UTR plus coding region containing the translation start site (5’-CTGCCTACCCACACTGAAGCCATA-3’). Control MO was previously described [21].

### RNA probe, mRNA preparation and whole-mount *in situ* hybridization

Synthesis of *Xenopus laevis* Aplnr, Erg, Etv2 and SpiB *in situ* hybridization probes was previously described [22–25]. *Xenopus laevis* PlexinD1 probe (Open Biosystem, EXL1051-432320) was linearized with Sal I and transcribed with T7 RNA polymerase. The open reading frame of *Xenopus laevis* Anxa3 was PCR amplified with Pfu turbo polymerase from a full-length clone (BC072890) and cloned into the EcoRV site of pBluescript SK(+) for probe generation and into the EcoRV site of pT7TS for mRNA synthesis. For probe synthesis, the plasmid was linearized with EcoRI and transcribed with T7. To generate Anxa3 synthesized capped mRNA, linearize the Anxa3-pT7TS plasmid with Eco RI and transcribe with T7 mMessage Machine (Ambion). GFP mRNA was generated from EGFP-pT7TS following linearization with SalI and transcription with T7 RNA polymerase (mMessage Machine, Ambion). The *Danio rerio* Anxa3a and b *in situ* probes (Open Biosystems, MDR1734-99822273) were transcribed with T7 RNA polymerase following linearization with Eco RV and SmaI, respectively. The full coding sequence plasmid of mouse Anxa3 (Open Biosystems, EMM1002-99866684) and partial sequence of PlexinD1 (Open Biosystems, MMM1013-66046) was used to generate RNA probe. Anxa3, linearize with NotI and transcribe with T3; PlexinD1, linearize with SalI and transcribe with T7.

Whole-mount *in situ* hybridization using digoxigenin-labeled RNA probes was performed using standard methods previously outlined [26]. The vasculature in experimental embryos were scored “normal” or “wild-type” for the following: continuous posterior cardinal vein (pcv), organized vascular plexus flank (vp) and aortic arches (aa), as well as presence of intersomitic vessels (isv); and scored “disrupted” when at least 3 of the following were observed: discontinuous pcv, unorganized vp and loss of aa or isv formation. For the Anxa3 MO1 rescue experiments, embryos were deemed “rescued” if they met the following criteria: presence of isv and aa, and a continuous pcv.

### Immunofluorescent antibody staining in *Xenopus*

Whole-mount immunofluorescent staining of *Xenopus laevis* embryos was conducted as previously outlined [27]. Embryos were fixed in MEMFA at room temperature for 1 hour. Rabbit anti-Claudin-5 polyclonal antibody (Santa Cruz Biotechnology, sc-28670) was used at a concentration of 1:200. Alexa Fluor 488 goat anti-rabbit secondary antibody (Life Technologies, A-11034) was used at 1:500. Immunofluorescently stained embryos were visualized in benzyl alcohol: benzyl benzoate (BABB), 1:2 and imaged using confocal microscopy.

### Cell culture based assays

All cell lines were obtained directly via ATCC (PCS-100-010, CRL-2279, CRL-2299, CRL-2581 and CRL-2161). Western blot analysis using rabbit anti-ANXA3 polyclonal antibody (Abnova, H00000306-D01), rabbit anti-CLAUDIN-5 polyclonal antibody (Santa Cruz Biotechnology, sc-28670), goat anti-VE-Cadherin polyclonal antibody (Santa Cruz Biotechnology, sc-6458), rabbit anti-ZO-1 polyclonal antibody (Life Technologies, 40–2200) and mouse anti-β-Actin monoclonal antibody (Cell Signaling, 3700) was carried out using standard protocols. For all *in vitro* experiments using siRNAs, 35 μM of control and Anxa3 siRNAs (SMARTpool: ON-TARGETplus, GE Dharmacon) were transfected using siPORT Amine (Ambion) for 72 hours prior to performing assays.

Scratch assays: confluent monolayers of HUVECs were scratched with a P200 pipet tip to create an EC-free zone, washed with PBS, imaged and incubated at 37°;5%-CO_2_. Cells were allowed to recover and “heal” for 24 hours, washed with PBS and imaged. At 0 and 24 hours post-scratch, the EC-free zones were outlined and the area was calculated using ImageJ. The differences in area between hour 0 and 24 hours were used to determine the area covered by the HUVEC in both control and Anxa3 siRNA treatments. Three separate experiments ran in quadruplicate were performed and used to verify statistical significance using an unpaired t-test and standard error of the mean, SEM (GraphPad Prism).

Cell proliferation and death was analyzed in siRNA treated HUVECs grown on round coverslips coated with 0.1% gelatin. After siRNA transfection (72 hours), coverslips were washed with PBS 3 times and fixed in 4% paraformaldehyde for 10 min at room temperature (RT). Next, cells were permeabilized in PBSN (0.1% NP-40 in PBS) for 15 min at RT and incubated in CAS-block (Life Technologies) for 30 min at RT. Rabbit anti-Phospho-Histone H3 polyclonal antibody (Millipore, 06–570) and rabbit anti-cleaved Caspase-3 polyclonal antibody (Cell signaling, 9661) antibodies were applied at 1:60 in CAS-block and incubated at RT for 1 hour. Coverslips were then washed 3 times in PBSN at RT and Alexa Fluor 555 secondary antibodies (Life Technologies) were applied at 1:200 for 1 hour at RT. Cells were then washed 3 times with PBSN and mounted onto slides using ProLong Gold antifade reagent plus DAPI (Life Technologies). Phospho-Histone H3 and cleaved Caspase-3, DAPI stained cells were counted and compared to the total number of DAPI cells. In four separate experiments, 3 different regions of triplicate siRNA treated HUVEC-coated coverslips were analyzed. Statistical significance was determined using an unpaired t-test and standard error of the mean, SEM (GraphPad Prism).

Immunofluorescent analysis of VE-Cadherin (Santa Cruz Biotechnology, sc-6458) and ZO-1 (Life Technologies, 40–2200) in HUVECs treated with control and Anxa3 siRNAs was performed as described above. Claudin-5 (Life Technologies, 34–1600) was immunofluorescently detected according to previously published work [28].

### Zebrafish and transgenic mice

The zebrafish embryos were provided from Dr. James Amatruda. VegfR2 null mouse embryos were generated by mating VegfR2-LacZ heterozygous males and females (provided by Drs. Janet Rossant and Eli Keshet).

### Ethics Statement

All animal studies were performed in accordance with UT Southwestern Medical Center Institutional Animal Care and Use Committee (IACUC) approved protocol APN 2007–0044, approval date January 20, 2011. Mice were euthanized by CO_2_ asphyxiation followed by cervical dislocation. *Xenopus laevis* males were euthanized by immersion in tricaine methane sulfonate solution (5g/L, MS222) followed by cervical sectioning and double pithing.

## Results

### Anxa3 is expressed in endothelial and myeloid cell lineages

**Fig 1:**
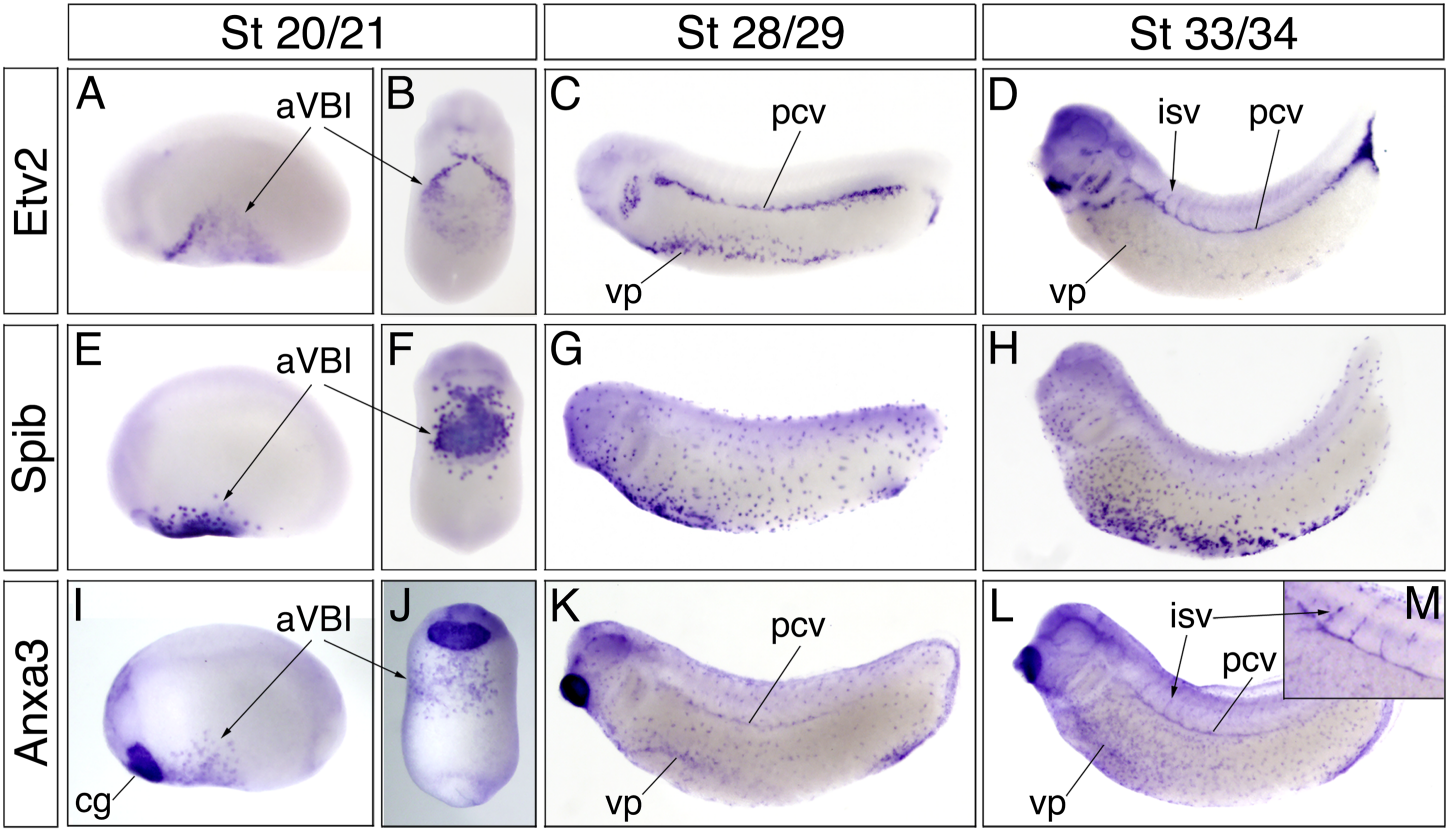
Anxa3 is expressed in endothelial and myeloid cells of developing *Xenopus laevis embryos*. Whole-mount *in situ* hybridization analysis of Etv2, SpiB and Anxa3 transcripts in *Xenopus* embryos at stages (St) 20/21, 28/29 and 33/34 (A-M). Lateral (A,C,D,E,G,H,I,K,L) and ventral (B,F,J) views show a close association of Anxa3 with the developing endothelial (Etv2) and myeloid (SpiB) lineages. At early stages (I,J), Anxa3 is expressed in the anterior ventral blood island (aVBI), which consists of adjacent Etv2 (A,B) and SpiB (E,F) expressing cells. At St 28/29 and 33/34, Anxa3 (K,L) is detected in the major vascular structures highlighted by Etv2 expression (C,D): the posterior cardinal vein (pcv), vascular plexus flank (vp) and intersomitic vessels (isv). (M) High magnification view of an embryo showing strong expression of Anxa3 in the pcv and isv. Anxa3 is also observed in the myeloid cells (marked by SpiB) that migrate throughout the entire embryo (compare the G,H to K,L). Throughout development, Anxa3 is also present in the cement gland (cg).

To better understand the function of Anxa3 during development, we first performed whole-mount *in situ* hybridization on *Xenopus laevis* embryos to identify tissues that express Anxa3. Comparison with the endothelial marker Etv2 and the myeloid marker SpiB showed that Anxa3 transcripts are expressed in both of these lineages (Fig 1). Anxa3 transcripts were detected around stage 18–20 when ECs and myeloid cells (MCs) are closely associated in the ventral blood island region (Fig 1A, 1B, 1E, 1F, 1I and 1J). Anxa3 expression remained in both cell populations throughout embryonic development as the location of the two cell types diverged, and was also detected in the facial cement gland (Fig 1C, 1D, 1G, 1H, 1K and 1L). Anxa3 was present in the developing vascular structures including the posterior cardinal veins, lateral vascular plexus flank and intersomitic vessels, as well as the MCs that migrate and disperse throughout the entire embryo.

**Fig 2:**
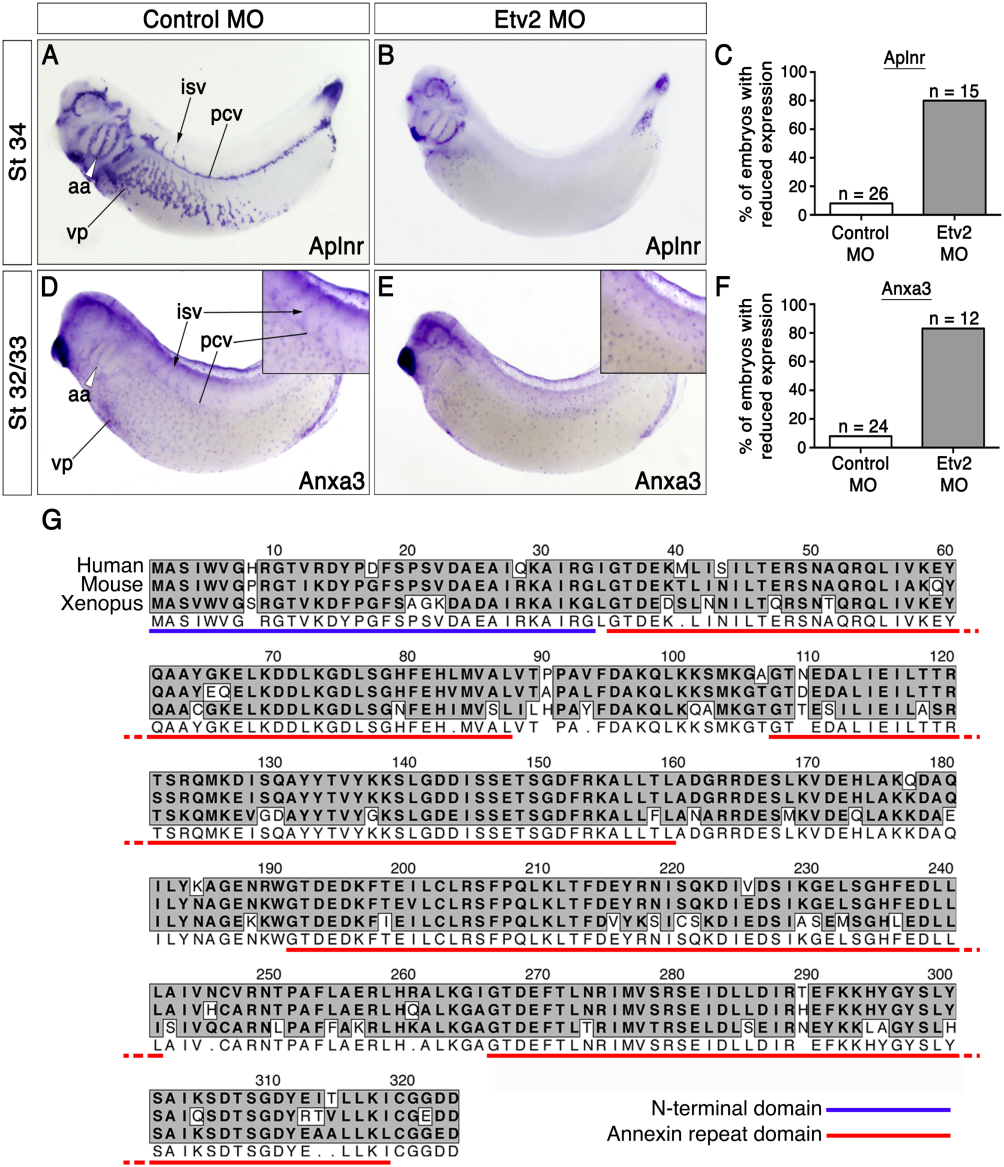
Anxa3 is expressed in Etv2-dependent endothelium. Analysis of Aplnr and Anxa3 expression in embryos injected with 25 ng of control (A,D) and Etv2 (B,E) MOs (stage 32–34, lateral views). (A,B) Etv2 MO blocks expression of the vascular marker Aplnr, as compared to control MO treated embryos. (C) Graphical representation of the percentage of control and Etv2 MO injected embryos showing reduced vascular expression of Aplnr. (D,E) Vascular expression of Anxa3 is lost in Etv2 MO injected embryos but retained in control MO injected embryos. Insets are higher magnification views centered on the developing posterior cardinal vein (pcv) and intersomitic vessels (isv). Notice that Anxa3 transcripts are present in myeloid cells in both control and Etv2 morphants. (F) Graphical representation of the percentage of control and Etv2 MO injected embryos showing reduced vascular expression of Anxa3. aa, aortic arches (white arrowhead); vp, flank vascular plexus. (G) Amino acid sequence alignment of human, mouse, *Xenopus* ANXA3 and zebrafish ANXA3(a) and ANXA3(b) proteins (MacVector—dark shading indicates identity and light shading indicates similarity of amino acids). The N-terminal domain is underlined in blue and the Annexin repeat domains (4) are underlined in red.

To further verify EC-enrichment of Anxa3, we performed experiments to generate embryos lacking vasculature. We accomplished this by knocking down the Etv2 gene using previously published Morpholino Oligonucleotides (MO), which is required for expression of virtually all endothelial genes [22]. Embryos injected with control and Etv2 MOs were analyzed by *in situ* hybridization for the presence of blood vessels. Etv2 MO-injected embryos showed a dramatic loss, and many times a complete absence, in endothelial expression of Anxa3 and the control EC marker, Apelin receptor (Aplnr), while expression of these genes was unaltered in control MO treated embryos (Fig 2A–2F). Transcript levels of Anxa3 were grossly normal in MCs, however this was expected given that Etv2 is not required for myeloid gene expression in *Xenopus* embryos [22]. Overall, these results showed that Anxa3 is expressed in both the myeloid lineage and Etv2-dependent endothelium throughout development.

### EC expression of Anxa3 is conserved throughout evolution

Amino acid alignment of ANXA3 revealed a high level of protein conservation among various vertebrate species (Fig 2G). The annexin repeat and N-terminal domains that comprise Anxa3 showed 85% and 88% amino acid identity between mice and frogs, respectively. This high sequence identity suggested that Anxa3 function is likely conserved among vertebrates.

**Fig 3:**
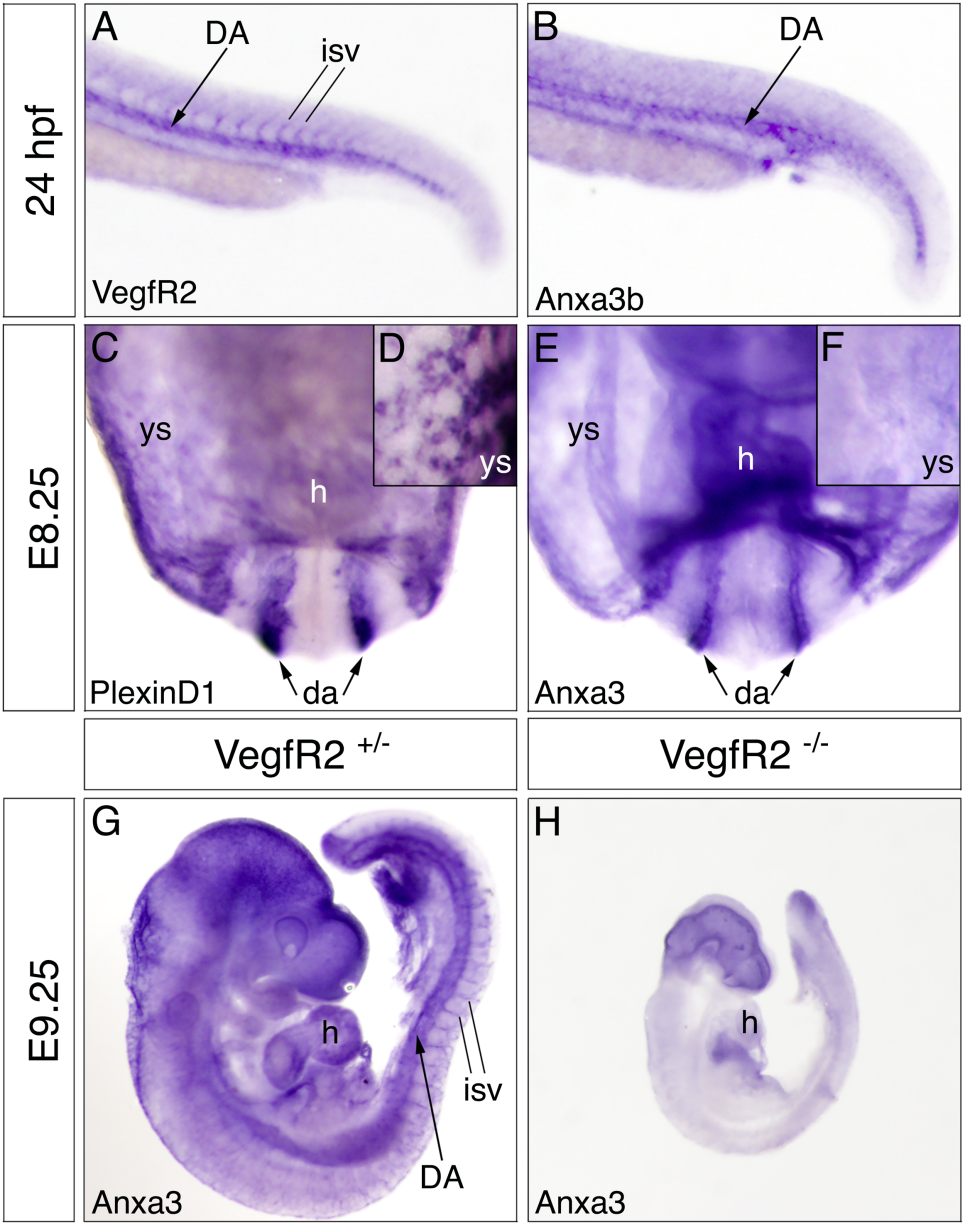
Expression of Anxa3 in the endothelial lineage is conserved across multiple species. (A,B) Whole-mount *in situ* hybridization analysis for vascular endothelial growth factor receptor 2 (VegfR2) and Anxa3b expression in *Danio rerio* embryos, 24 hours post fertilization (hpf). Close-up, lateral views of the tail regions are shown. Anxa3b transcripts are restricted to the developing dorsal aorta (DA), whereas VegfR2 is also expressed in the intersomitic vessels (isv). (C-F) Embryonic (E) day 8.25 mouse embryos were assayed for Anxa3 and the vascular marker PlexinD1 by whole-mount *in situ* hybridization (anterior views). Similar to PlexinD1, Anxa3 is observed in the paired dorsal aortae (da, arrows) and heart (h) region of the embryo. Anxa3 is absent in the extra-embryonic vessels of the yolk sac (ys) that are marked by PlexinD1 expression. This is highlighted in close-up views of ys from PlexinD1 and Anxa3 stained E8.25 embryos (D,F). (G,H) E9.25 heterozygous (^+/-^) and homozygous (^-/-^) VegfR2 embryos analyzed for Anxa3 expression by *in situ* hybridization (lateral views). Anxa3 is detected in the dorsal aorta (DA), intersomitic vessels (isv) and heart (h) region of VegfR2^+/-^ embryos, while VegfR2^-/-^ null embryos, which lack all blood vessels, displayed no observable vascular staining of Anxa3 (background staining is detected in the head and just outside the heart region).

Using whole-mount *in situ* hybridization, we found that similar to frogs, ECs of developing zebrafish and mouse embryos express Anxa3 RNA (Fig 3). Although we were unable to detect Anxa3a transcripts in zebrafish (data not shown), Anxa3b expression was limited to the ECs in the dorsal aortae (compare Fig 3A and 3B). In mice, Anxa3 transcripts were abundant in the heart region (endocardium) and in blood vessels throughout the embryonic (E) day 8.25 embryo (Fig 3C and 3E). However Anxa3 was not expressed in the extra-embryonic blood vessels of the yolk sac, as demarcated by the endothelial marker PlexinD1 (Fig 3D and 3F). Anxa3 expression persisted in developing blood vessels including the dorsal aorta and intersomitic vessels at E9.25, and the large head vessels of E13.5 embryos (Fig 3G and data not shown). Analysis of VEGF receptor 2 (VEGFR2) mutant mice [29], which fail to form all blood vessels, showed a near complete loss of Anxa3 transcripts confirming endothelial expression (Fig 3H). In all, these expression studies suggest a significant and conserved role for Anxa3 during vascular development.

### Anxa3 influences EC migration *in vitro*

**Fig 4:**
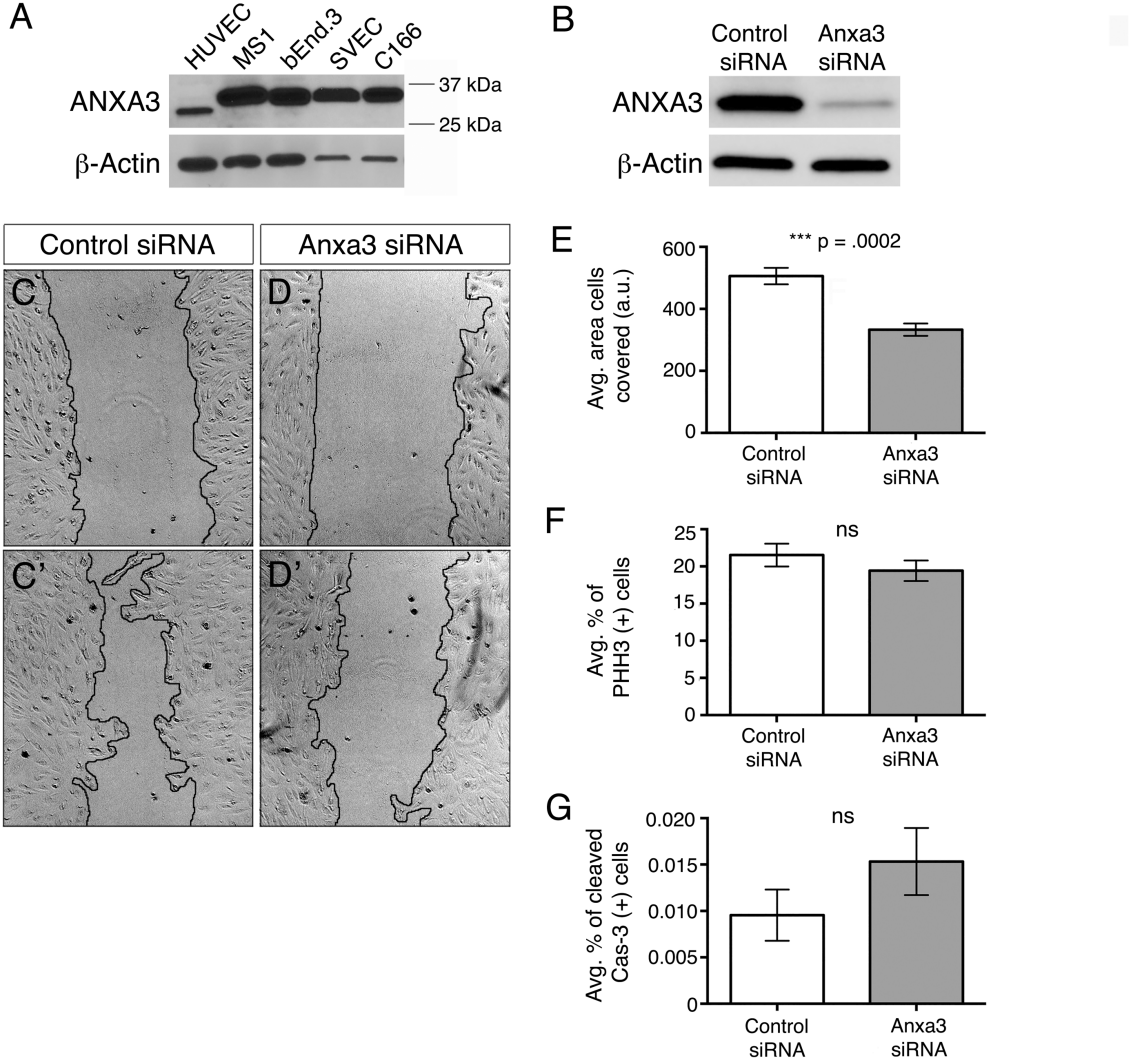
Loss of Anxa3 function *in vitro* results in aberrant cellular behavior. (A) Western blot analysis of ANXA3 protein levels in human (HUVEC) and mouse endothelial cell lines (MS1, bEnd.3, SVEC and C166). The ANXA3 antibody consistently recognized a protein in HUVEC, at the approximate predicted size (36 kDa), that was slightly smaller than mouse ANXA3. β-Actin was used as a loading control. (B) Western blot analysis of ANXA3 in HUVECs treated with control and Anxa3 siRNAs (35 μM) showed dramatic reduction in Anxa3 protein levels as normalized to β-Actin. (C-D’) Scratch assay results of control (C,C’) and Anxa3 (D,D’) siRNA treated HUVECs at 0 (C,D) and 24 (C’,D’) hours post scratch. EC-free areas are outlined in black. (E) Quantification of the EC-free area within the scratch assays, as determined by ImageJ in arbitrary units (a.u.). Note the significantly reduced repopulation of the wound by Anxa3 siRNA treated ECs. (F,G) Control and Anxa3 siRNA treated HUVECs were analyzed for rates of cell proliferation and death by immunofluorescent staining with Phospho-Histone H3 (PHH3) and Cleaved Caspase-3 (Cas-3), respectively, and presented in graphical form. No significant (ns) changes were observed. (E-G) Data are presented as the standard error of the mean, SEM.

Similar to embryonic ECs, Anxa3 protein and mRNA were present at substantial levels in a number of EC lines (Fig 4A and data not shown). To test whether Anxa3 affects EC behaviors, human umbilical vein endothelial cells (HUVEC) were transfected with siRNAs targeted to Anxa3 and subjected to various assays *in vitro*. Initial western blot analysis indicated that Anxa3 siRNAs efficiently reduced levels of Anxa3 protein (Fig 4B). Control and Anxa3 siRNA treated HUVECs were used in standard “scratch” (or wound healing) assays in which a defined region of confluent cells are scraped away and EC migration is assessed via extent of repopulation of the “wound” area. Over a period of approximately 24 hours, ECs transfected with control siRNAs displayed substantial migration into the wounded area, while coverage of the scratch with Anxa3 depleted ECs was significantly reduced (Fig 4C–4E). In addition, the Anxa3 siRNA-induced migratory defects were not due to changes in cell proliferation and death, as control and Anxa3 siRNA treated HUVECs exhibited comparable rates of proliferation and apoptosis (Fig 4F and 4G). Taken together, we conclude that Anxa3 influences EC migratory behavior *in vitro*.

### EC coalescence and vessel morphogenesis is regulated by Anxa3

**Fig 5:**
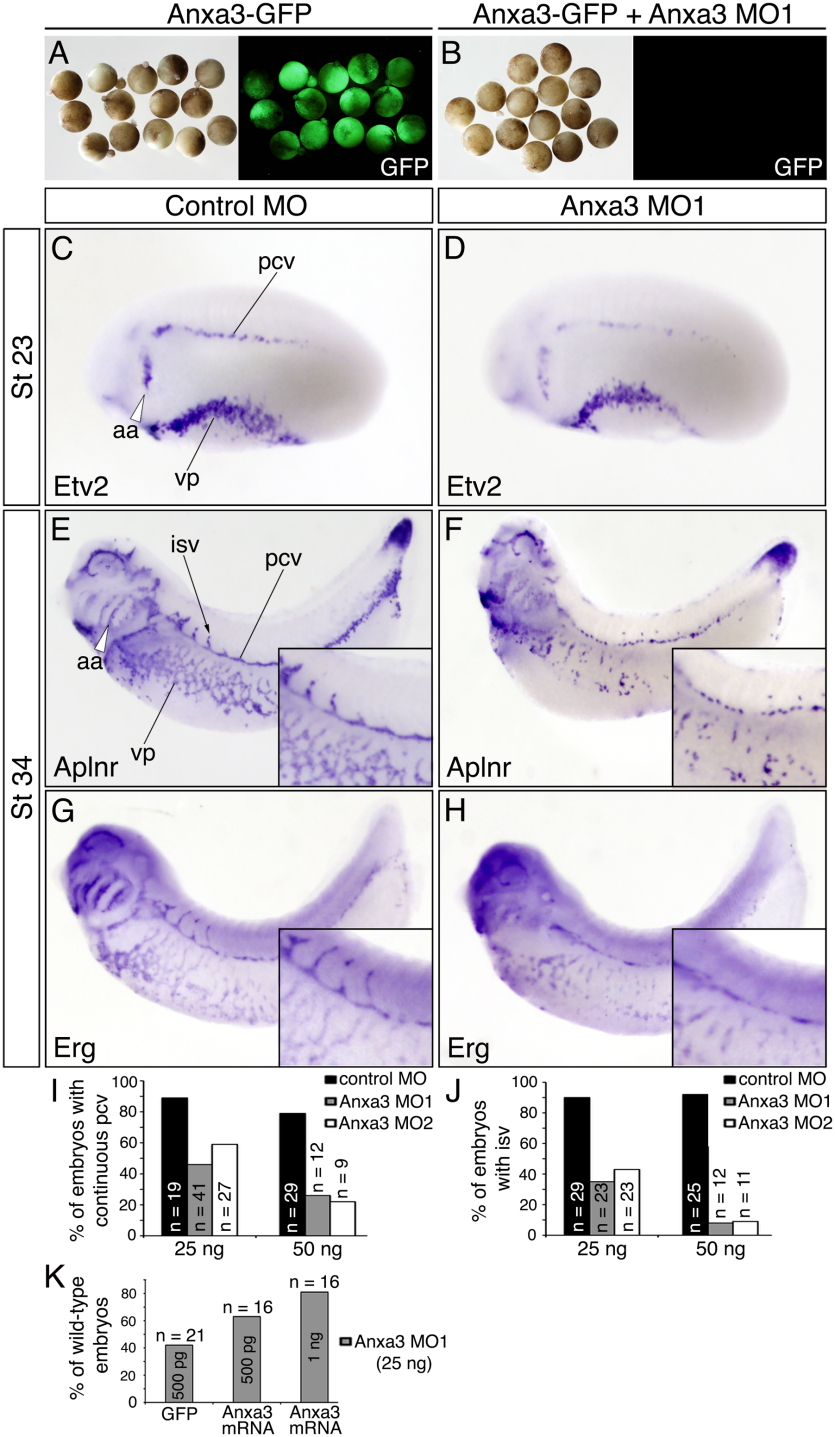
Anxa3 MO treated embryos fail to undergo proper blood vessel morphogenesis. (A,B) Anxa3 MOs efficiently block translation of a control transcript consisting of the Anxa3 5’UTR fused to the coding region of green fluorescent protein (GFP). Injection of this mRNA results in GFP expression in the embryo, however co-injection with Anxa3 MO blocks GFP translation. (C-H) Whole-mount *in situ* hybridization analysis (lateral views) on control MO and Anxa3 MO1 (25 ng) injected embryos. (C,D) At stage (St) 23, control and Anxa3 MO treated embryos show grossly similar expression levels of the EC differentiation marker Etv2. (E-H) At St 34, major vascular defects were visualized by *in situ* hybridization for Aplnr and Erg expression. Anxa3 morphants display non-continuous vessels and rarely form intersomitic vessels (isv) or aortic arches (aa). Insets show high magnification views of the posterior cardinal vein (pcv) and isv region. Notice in some cases (F), ECs appear unattached but aligned in the proper location. (I, J) Categorization of two non-overlapping Anxa3 MO (MO1 and 2) phenotypes compared to control MO treated embryos (St 34). Embryos injected with either non-overlapping Anxa3 MO seldom form isv and continuous, organized pcv. n, number of embryos assayed. (M) Anxa3 MO1-induced phenotypes (discontinuous pcv, unorganized vp and loss of isv and aa), as assessed by *in situ* hybridization for Aplnr transcripts (S2 Fig) and presented in graphical form, can be rescued in a dose dependent manner by co-injection of Anxa3 mRNAs.

To test whether Anxa3 is required for blood vessel formation during development, two non-overlapping antisense MOs (MO1 and 2), targeted to the 5’ untranslated region (UTR), were used to suppress Anxa3 translation in the embryo. A control mRNA construct containing the 5’ UTR plus 42 nucleotides of coding sequence of Anxa3 was fused to the coding region of green fluorescent protein (GFP) and injected into 1-cell staged embryos to test knock down efficiency of the MOs. Injection of mRNA alone resulted in expression of GFP, while no GFP was detected in embryos co-injected with either MOs (Fig 5A and 5B and data not shown) suggesting high binding efficiency of the Anxa3 MOs to target sequences.

Next, control and Anxa3 MOs were injected into 1 cell of the 2-cell staged embryo at different doses and analyzed by whole-mount *in situ* hybridization for expression of various vascular markers (Table 1). Each cell of the 2-cell stage embryo will give rise to one half of the entire embryo, thus the uninjected side of the embryo serves as an internal, developmental control. At early embryonic stages when the major vessels are just beginning to form, we observed no obvious, qualitative defects in the developing vasculature, as assessed by the expression of the blood vessel markers Etv2 and Aplnr, and by pattern of the posterior cardinal veins (PCV), ventral vascular plexus (VP) and aortic arches (Fig 5C and 5D, Table 1 and S1 Fig). However, by stages 32–34, prominent vascular defects were easily identified in Anxa3 morphants as compared to control MO injected embryos (Fig 5E–5H, 5K, 5L and S1 Fig). For example, ECs in the VP appeared as unorganized and scattered single ECs compared to the normally cohesive and stereotyped plexus of fine vessels (Fig 5E–5H). The PCVs were often discontinuous and incomplete (Fig 5F, 5H and 5I). Although ECs aligned at the regions where the PCVs normally form, they seldom assembled into the smooth vessel that normally forms in control MO treated embryos (Fig 5E and 5G). In fact, similar to the vascular plexus, the defective PCVs consisted of individual and groups of ECs that did not coalesce into a continuous vessel. These PCV defects are the likely reason why intersomitic vessels, which sprout off the PCV (Fig 5E and 5G), rarely formed in Anxa3 treated embryos (Fig 5F, 5H, 5J and S1A–S1D Fig).

**Table 1:** Inhibition of ANXA3 function leads to vascular disruptions.

St 24–26				
Marker	Treatment	Normal vasculature	Disrupted vasculature	Total no. embryos
*Aplnr*	Control MO (25 ng)	91%	9%	11
	Anxa3 MO1 (25 ng)	93%	7%	14
*Etv2*	Control MO (25 ng)	96%	4%	23
	Anxa3 MO1 (25 ng)	100%	0%	20
St 34				
Marker	Treatment	Normal vasculature	Disrupted vasculature	Total no. embryos
*Aplnr*	Control MO (25 ng)	87%	13%	55
	Control MO (50 ng)	84%	16%	25
	Anxa3 MO1 (25 ng)	26%	74%	49
	Anxa3 MO1 (50 ng)	8%	92%	12
	Anxa3 MO2 (25 ng)	50%	50%	24
	Anxa3 MO2 (50 ng)	9%	91%	11
*PlexinD1*	Control MO (25 ng)	100%	0%	16
	Anxa3 MO1 (25 ng)	18%	82%	22
	Anxa3 MO2 (25 ng)	45%	55%	11
*Erg*	Control MO (25 ng)	92%	8%	13
	Anxa3 MO1 (25 ng)	20%	80%	15

To demonstrate specificity of the Anxa3 MOs and the subsequent phenotypes, we performed mRNA rescue experiments. Capped mRNAs encoding GFP and Anxa3, but lacking the MO target sequences, were co-injected with Anxa3 MOs into 1 cell of the 2-cell staged embryo and vascular disruptions were assessed using *in situ* hybridization for Aplnr. Co-injection of GFP had negligible rescue activity (Fig 5K, S2A and S2C Fig), whereas a combination of Anxa3 mRNAs and MOs resulted in a dose dependent rescue of normal blood vessel formation (Fig 5K, S2B and S2D Fig). We conclude that the phenotypes observed in these studies were due to a specific reduction in Anxa3 protein, and reveal a critical role for Anxa3 in early blood vessel morphogenesis.

### Blood vessel formation is unaltered in the presence of excess Anxa3

**Fig 6:**
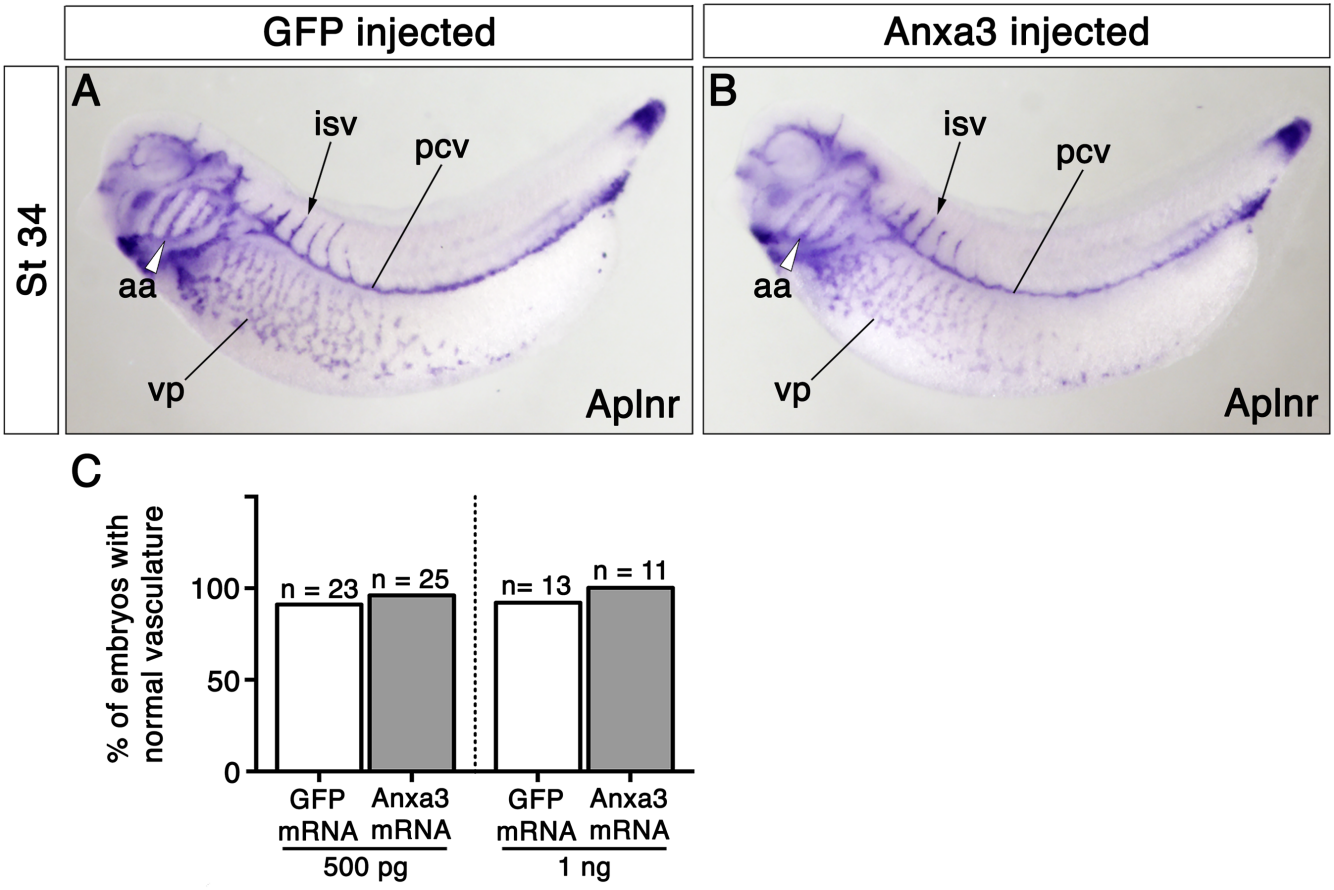
Anxa3 gain-of-function has no effect on developing blood vessels *in vivo*. (A,B) Whole-mount *in situ* hybridization for Aplnr expression in GFP and Anxa3 mRNA injected embryos (stage 34, lateral views). No changes in overall vessel morphology or levels of Aplnr expression were observed. (C) The percent (%) of GFP and Anxa3 mRNA injected embryos displaying normal vascular formation, as assessed by Aplnr expression, are shown. Aortic arches, aa; intersomitic vessel, isv; n, number of embryos assayed; posterior cardinal vein, pcv and vascular plexus, vp.

We next tested whether excess Anxa3 was sufficient to induce changes in formation of the embryonic vasculature. Anxa3 mRNAs, similar to the MO rescue experiments (Fig 5K), were injected into 1 cell of 2-cell staged embryos at a range of doses and analyzed by whole-mount *in situ* hybridization. Blood vessel formation was unaffected, as expression of Aplnr and overall vessel morphology in both the injected and uninjected sides of GFP and Anxa3 mRNA treated embryos at stages 26 and 34 were normal (Fig 6A–6C and S3 Fig). In addition, transfection of plasmids encoding Anxa3-GFP and Anxa3-HA fusion proteins had no adverse effects on cell morphology or gene expression of various markers in MS1 mouse ECs in culture (data not shown). These results demonstrated that although Anxa3 is required for proper formation of blood vessels, over-expression of Anxa3 does not alter vascular development.

### Loss of Anxa3 impairs EC-EC junctions *in vivo* but not *in vitro*

The lack of assembly of ECs into continuous vessels in embryos treated with Anxa3 MOs indicated a potential defect in cell-cell contact. For example, loss of cellular adhesion between ECs could explain why vessels in Anxa3 MO treated embryos rarely formed smooth and continuous structures. To test this hypothesis, we performed whole-mount immunofluorescent staining on control and Anxa3 MO injected embryos for the EC-EC tight junction marker Claudin-5. We note that this particular adhesion component was used because a comprehensive screen of available antibodies only yielded recognition of Claudin-5 in *Xenopus laevis*. Using high-resolution confocal microscopy, we observed a strong signal for Claudin-5 in the PCV and ISVs of stage 34 control morphants (S4A Fig). Claudin-5 was also detected in fine neurons extending ventrally from the spinal cord, which was expected given previous data showing its expression in embryonic neural tissues [30]. Claudin-5 expression in the uninjected side of Anxa3 treated embryos was similar to controls, with signal detectable in the PCV, ISVs and neural cells from the spinal cord (S4B Fig). Conversely, on the Anxa3 MO injected side, Claudin-5 expression was either absent or severely diminished in the region where these vascular structures are normally found, yet it was still detected at normal levels in neural cells (S4C Fig).

To further explore a link between Anxa3 and Claudin-5 in mammalian EC-EC contacts, we examined Claudin-5 protein levels and localization in HUVECs treated with control and Anxa3 siRNAs. Interestingly, we observed no noticeable changes in Claudin-5 expression or cell membrane distribution in control and Anxa3 siRNA treated HUVECs (S4D, S4E and S4K Fig). Furthermore, the tight junction protein, ZO-1, and the adherens junction protein, VE-Cadherin, showed no detectable differences (S4F–S4I, S4L and S4M Fig). Similar results were obtained in a mouse brain endothelial cell line (bEnd.3, data not shown). These data demonstrated that knockdown of Anxa3 had little effect on EC-EC junctions in cultured mammalian ECs, yet Claudin-5 expression was disrupted in frog embryos. At this point, it remains unclear whether loss of Claudin-5 in *Xenopus* is a cause or consequence of the vascular defects present in Anxa3 depleted embryos.

### Multiple Annexins are expressed in the endothelial lineage of mammals

Given the disparity in the results of Claudin-5 expression *in vivo* and *in vitro*, we postulated that redundant functions of Annexins in mammalian ECs could explain these observations. In fact, in addition to Anxa3 and Anxa5 being present in mouse embryonic blood vessels (Fig 3E and 3F) [13], we found that Anxa2 and Anxa6 are expressed in the early embryonic endothelium of mice (S5A and S5B Fig). Moreover, various Annexin genes, such as Anxa1,2,3,5, and 6 are expressed in HUVECs (data not shown). Conversely, whole-mount *in situ* hybridization revealed that only Anxa2, not Anxa5 or Anxa6, were present in *Xenopus* embryonic blood vessels (S5C and S5D Fig and Xenbase). It is also interesting to note that Anxa1,2,4,5,6,11,13 are absent from developing blood vessels in zebrafish [31], thus distinguishing Anxa3b as the only Annexin gene identified in the fish vasculature to date (Fig 3B). Overall, these data indicate that, in contrast to frogs and fish, various Annexins are present in the mammalian vasculature and suggests potential overlapping or compensatory functions of Annexin family members in higher vertebrates.

## Discussion

In this report, we assess Anxa3 in the developing vertebrate vasculature. We demonstrate conservation of Anxa3 vascular expression across species and identify a novel role for Anxa3 in the endothelium, including an essential role during early development of the amphibian vasculature. Our findings forward understanding of annexins during embryonic tissue formation.

### Expression of Anxa3 is conserved in the developing endothelial lineage

Here, we characterize the early developmental expression profile of the *Xenopus laevis*, *Danio rerio* and *mus musculus* Anxa3 genes. We find that Anxa3 is present in both the endothelial and myeloid cells of frog embryos, starting as early as stage 18 when both cell populations are located adjacently within the ventral blood island (Fig 1). Anxa3 transcripts were later detected in the major vascular structures of the embryo, including the dorsal aorta, posterior cardinal veins and intersomitic vessels. Endothelial expression of Anxa3 was absent in embryos lacking blood vessels, confirming vascular specificity (Fig 2). The very high sequence identity of Anxa3 proteins in different species (Fig 2G) and conserved expression in the major blood vessels of fish and mice embryos (Fig 3) strongly suggest an important evolutionary function for Anxa3 during vascular development.

### Anxa3 regulates EC motility *in vitro*

To better understand the role of Anxa3 in vascular biology, we treated cultured human ECs (HUVEC) with siRNAs targeted against Anxa3 and assessed their ability to migrate using a standard cell “scratch” assay. We found that ECs depleted of Anxa3 were unable to fully recover and repopulate the wounded area as compared to control siRNA treated HUVECs (Fig 4C–4E). These results are in line with previous experiments whereby conditioned media from HEK 293 cells stably expressing Anxa3 induced HUVEC migration in Boyden chamber assays [20]. In addition, it was shown that excess Anxa3 promoted EC tube formation in Matrigel assembly experiments. Collectively, these studies substantiate a role for Anxa3 in controlling mammalian EC dynamics, including cell motility. However, the mechanisms by which Anxa3 regulate different cellular behaviors remains to be determined and will be a central question for understanding Anxa3 biology.

### Vasculogenic defects are associated with loss of Anxa3 function

The morpholino knockdown experiments conducted in this study demonstrated a requirement for Anxa3 during early vasculogenic development. Loss of Anxa3 caused severe vascular phenotypes characterized by isolated ECs and discontinuous blood vessels. In some instances Anxa3 morphants exhibited a reduction in EC numbers. Although early vascular formation appeared unperturbed, it is possible that the onset of vascular defects, at least in part, resulted from changes in cell death and/or proliferation. Although our *in vitro* siRNA studies showed no changes in either of these processes upon loss of Anxa3 function, we cannot rule out the possibility that changes in cell death and/or proliferation contribute to the Anxa3 MO phenotype.

Overall however, the observed vascular phenotypes implied possible defects in EC-EC adhesion rather than in specification, differentiation or guidance, as ECs expressed various specification and differentiation markers, and correctly localized where vessels normally form, yet remained as separate cells (Fig 5 and Table 1). To test this possibility, we surveyed vascular antibody cross-species reactivity in *Xenopus*, including VEGFR2, PECAM, VE-Cadherin, Endomucin, Isolectin-IB4 and tight junction protein Claudin-5, and found that only anti-Claudin-5 could be detected and used to visualize *Xenopus* vessels. Immunofluorescent microscopy therefore allowed us to assess EC-EC tight junctions using anti-Claudin-5. Unlike control MO injected embryos, endothelial Claudin-5 was largely missing in Anxa3 MO injected embryos, but retained in neural tissues (S4 Fig), suggesting a possible Anxa3 function in endothelial adhesion. However, it is unclear whether loss of Claudin-5 is a result of the loss of regulation by Anxa3 or rather the consequence of severely disrupted blood vessel morphogenesis. Further studies will be key to uncovering a potential Anxa3-Claudin-5 relationship *in vivo*.

By contrast, loss of Anxa3 function seemed to have no effect on junctional Claudin-5, ZO-1 or VE-Cadherin expression and localization *in vitro*. Interestingly, our embryonic expression studies (S5 Fig), along with previously published work [13], have shown that multiple Annexin family members are expressed in the developing vasculature of mammals in contrast to *Xenopus* and zebrafish embryos that have only one or a few endothelial Annexins (S5 Fig), [31]. This could explain the different results in Claudin-5 sensitivity to Anxa3 loss, given the increased likelihood of redundant functions in mammalian blood vessels. Future studies aimed at characterizing EC behavior upon loss of Anxa3 in mouse models will be of particular interest, as tools are in hand to examine more closely cell adhesion during vasculogenesis and angiogenesis in comparison to the phenotypes observed in Anxa3 morphants.

Alternatively, it is conceivable that the role of Anxa3 on cell adhesion is less important than its role in EC migration. Migration, including formation and extension of lamellipodia and filopodia, is critical for both cord formation and angiogenic sprouting, both processes impaired in Anxa3 MO treated embryos. This would fall in line with the migration defects observed *in vitro*, and could explain why individual ECs are observed in Anxa3 morphants (perhaps via disruption in their ability to extend and migrate towards each other) and why they fail to form vascular cord structures and ultimately blood vessels.

Overall, our investigation into the developmental role of Anxa3 revealed a critical function for Anxa3 during blood vessel morphogenesis. These experiments are the first to describe the role of Anxa3 *in vivo*, further implicating annexin proteins in endothelial biology and vascular disease.

## Supporting Information

S1 Fig Loss of Anxa3 leads to blood vessel disruption in *Xenopus* embryos. (A-D) Whole-mount *in situ* hybridization for Aplnr (A,B) and PlexinD1 (C,D) expression in control and Anxa3 MO1 (25 ng) treated frog embryos at stage (St) 25/26 and St 34, respectively (lateral views). Insets in C and D show high magnification views of the posterior cardinal vein (pcv) and intersomitic vessel (isv) region. Notice at St 25/26 the developing vasculature in Anxa3 morphants is comparable to control MO injected embryos, however by St 34, loss of Anxa3 results in major blood vessel defects in the aortic arches (aa), isv, pcvflank vascular plexus (vp), isv and flank vascular plexus (vp).pcv.
(TIF)

S2 Fig Anxa3 mRNA rescues Anxa3 MO-induced phenotypes. (A-D) Whole-mount *in situ* hybridization for Aplnr expression in embryos injected with Anxa3 MO1 plus GFP or Anxa3 MO1 plus Anxa3 mRNA (stage 34, lateral views). Note that the Anxa3 mRNA does not contain the Anxa3 MO target sequences. Embryos were categorized as “rescued” (D) by the presence of intersomitic vessels (isv, arrows), aortic arches (aa, white arrowheads), continuous posterior cardinal vein (pcv) and an organized vascular plexus (vp).
(TIF)

S3 Fig Over-expression of Anxa3 does not disrupt early blood vessel formation. (A,B) *In situ* hybridization analysis for Aplnr transcripts in embryos injected with 1 ng of Anxa3 mRNA (stage 28, lateral views). Formation of the aortic arches (aa), posterior cardinal vein (pcv) and flank vascular plexus are nearly identical in the uninjected (A) and Anxa3 mRNA injected (B) sides of frog embryos.
(TIF)

S4 Fig Anxa3 knock down leads to reduction of Claudin-5 protein in frogs but not *in vitro*. (A) Injected side of a control MO treated embryo (stage 34) immunofluorescently labeled for Claudin-5. Notice expression in the developing posterior cardinal vein (pcv), intersomitic vessels (isv) and neurons (n) extending from the spinal cord. (B) A stage 33, uninjected side of an Anxa3 MO treated embryo, stained for Claudin-5, shows similar levels and localization of Claudin-5 protein compared to A. (C) Immunofluorescent detection of Claudin-5 in the Anxa3 MO injected side of the embryo in B. Note the lack of Claudin-5 in the developing pcv and isv region (large arrowhead), yet expression is present in neurons (n). (D-M) Human umbilical vein endothelial cells (HUVEC) treated with control and Anxa3 siRNAs (35 μM) and analyzed for the tight junction markers, Claudin-5 and ZO1, and the adherens junction marker, VE-Cadherin, via immunofluorescent staining (D-I) and western blot analysis, including ANXA3 (J-M). No detectable changes were observed. Predicted protein sizes: CLAUDIN-5, 23 kDa; ZO-1, 220 kDa; VE-Cadherin, 94 kDa. Note that the CLAUDIN-5 antibody detects two bands at approximately 23 kDa and slightly smaller than 20 kDa).
(TIF)

S5 Fig Multiple annexins are expressed in the embryonic blood vessels of mouse and *Xenopus*. (A-D) Whole-mount *in situ* hybridization analysis of Anxa2 and Anxa6 transcripts in *Xenopus* embryos (lateral views) at stages (St) 34–35 and mouse embryos (ventral views) at embryonic (E) day 8.25. (A) Mouse Anxa2 is weakly expressed in the paired dorsal aortae (da, white arrowheads) that lie underneath the Anxa2 expressing somites (s). The da and somites on the right side of the embryo are outlined in white and black, respectively. Anxa2 transcripts can also be detected in the blood vessels of the extra-embryonic yolk sac (ys), inset. (B) Anxa6 RNA is strongly observed in the ys vasculature and the paired da. (C,D) In the frog embryo, Anxa2 transcripts are detected in the intersomitic vessels (isv), posterior cardinal vein (pcv) and aortic arches (aa), while Anxa6 transcripts are not observed in developing blood vessels.
(TIF)
