# Supplementary figures and images for: Annexin A3 Regulates Early Blood Vessel Formation
Source: PLoS One. 2015 Jul 16;10(7):e0132580. doi: 10.1371/journal.pone.0132580 (PMC4504506; doi:10.1371/journal.pone.0132580)

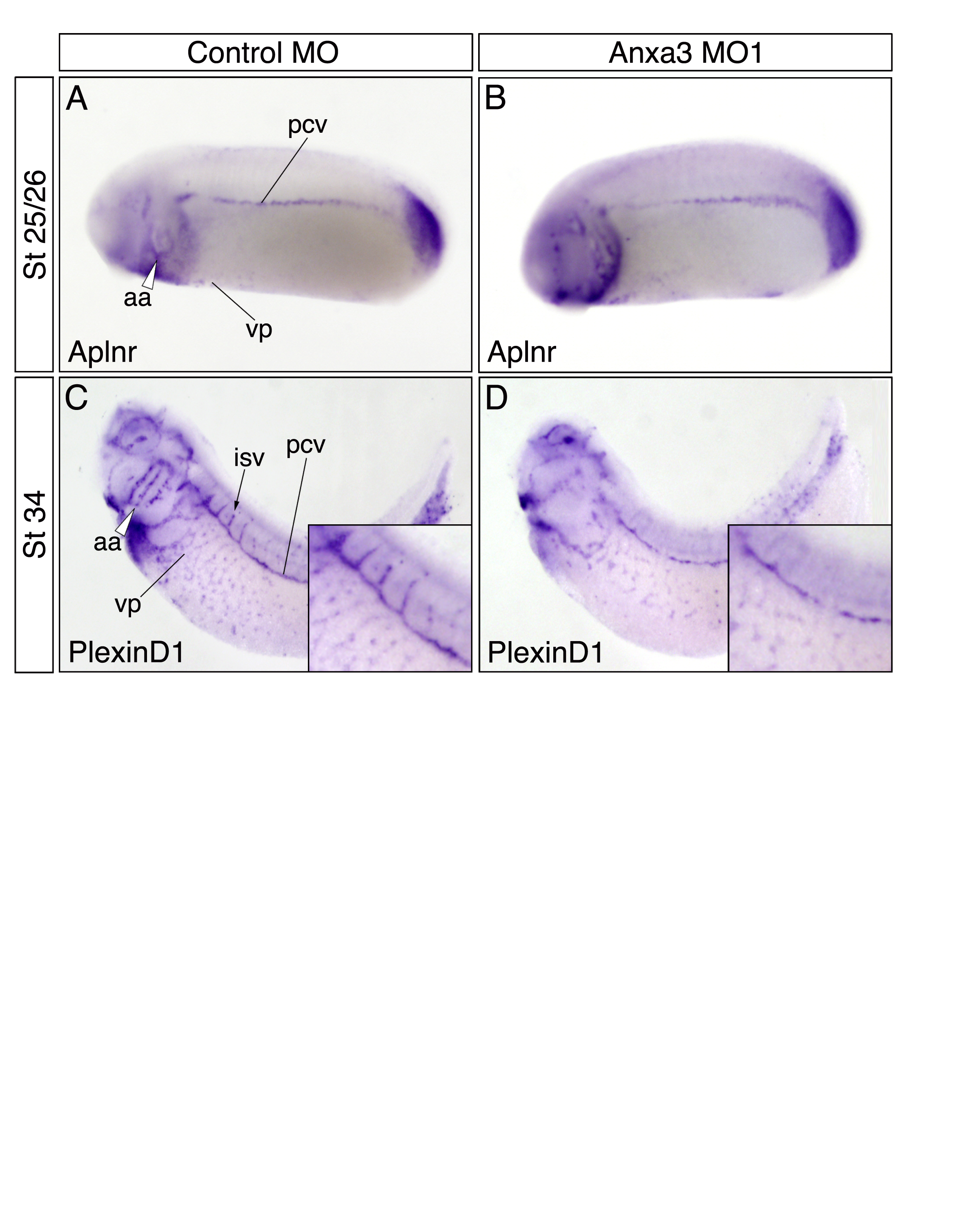

Supplement: S1 Fig — (A-D) Whole-mount in situ hybridization for Aplnr (A,B) and PlexinD1 (C,D) expression in control and Anxa3 MO1 (25 ng) treated frog embryos at stage (St) 25/26 and St 34, respectively (lateral views). Insets in C and D show high magnification views of the posterior cardinal vein (pcv) and intersomitic vessel (isv) region. Notice at St 25/26 the developing vasculature in Anxa3 morphants is comparable to control MO injected embryos, however by St 34, loss of Anxa3 results in major blood vessel defects in the aortic arches (aa), isv, pcvflank vascular plexus (vp), isv and flank vascular plexus (vp).pcv. (TIF) [file pone.0132580.s001.tif]

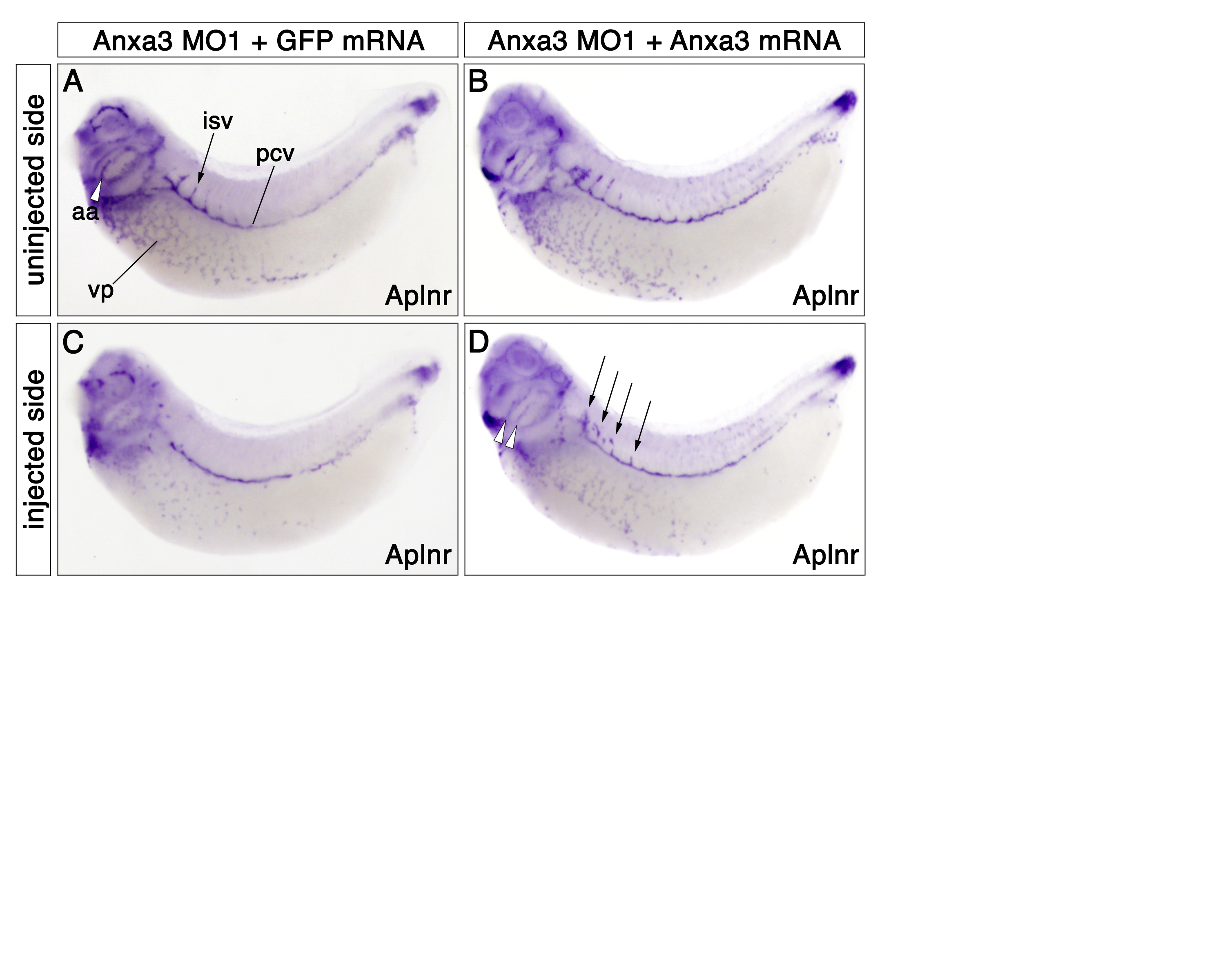

Supplement: S2 Fig — (A-D) Whole-mount in situ hybridization for Aplnr expression in embryos injected with Anxa3 MO1 plus GFP or Anxa3 MO1 plus Anxa3 mRNA (stage 34, lateral views). Note that the Anxa3 mRNA does not contain the Anxa3 MO target sequences. Embryos were categorized as “rescued” (D) by the presence of intersomitic vessels (isv, arrows), aortic arches (aa, white arrowheads), continuous posterior cardinal vein (pcv) and an organized vascular plexus (vp). (TIF) [file pone.0132580.s002.tif]

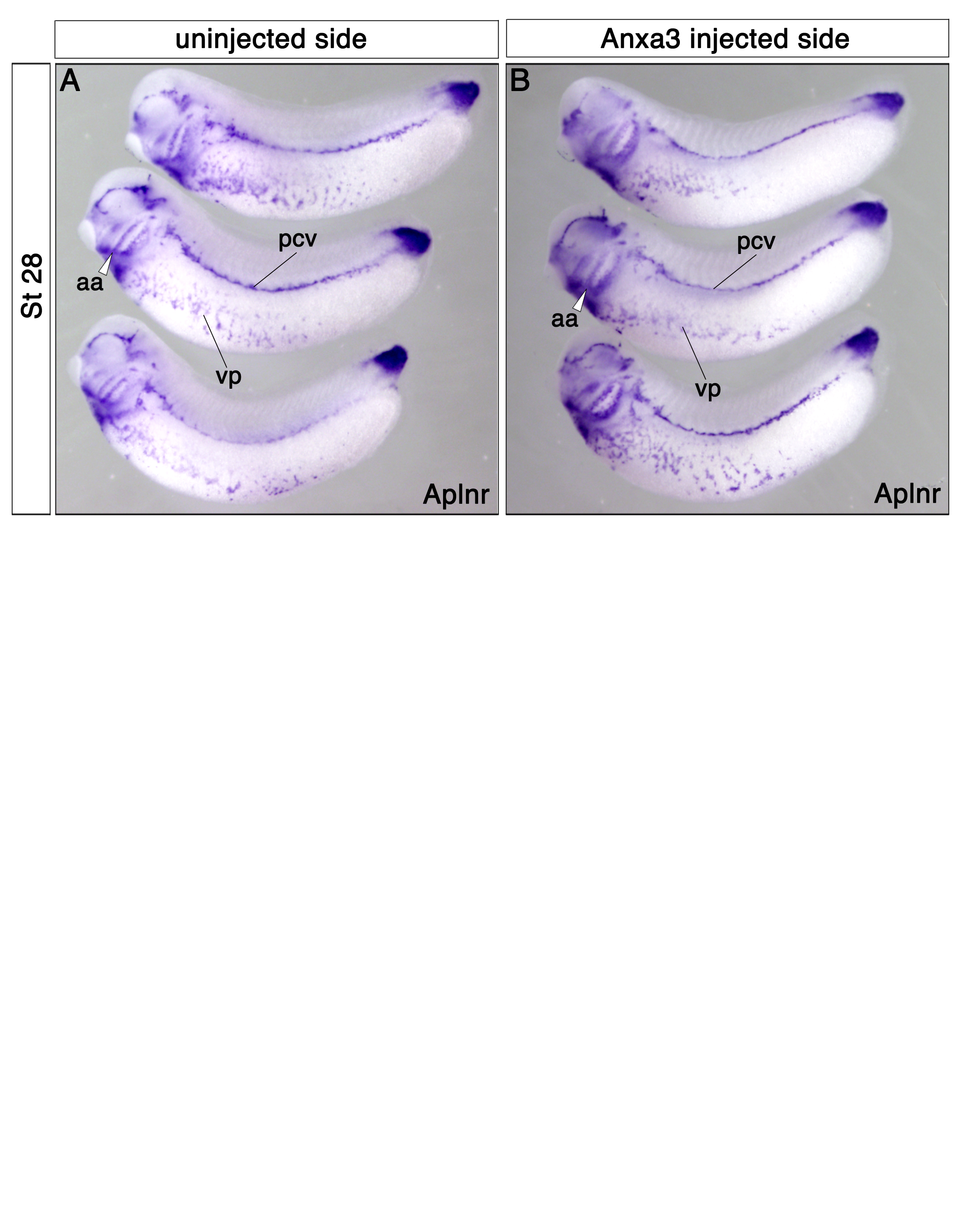

Supplement: S3 Fig — (A,B) In situ hybridization analysis for Aplnr transcripts in embryos injected with 1 ng of Anxa3 mRNA (stage 28, lateral views). Formation of the aortic arches (aa), posterior cardinal vein (pcv) and flank vascular plexus are nearly identical in the uninjected (A) and Anxa3 mRNA injected (B) sides of frog embryos. (TIF) [file pone.0132580.s003.tif]

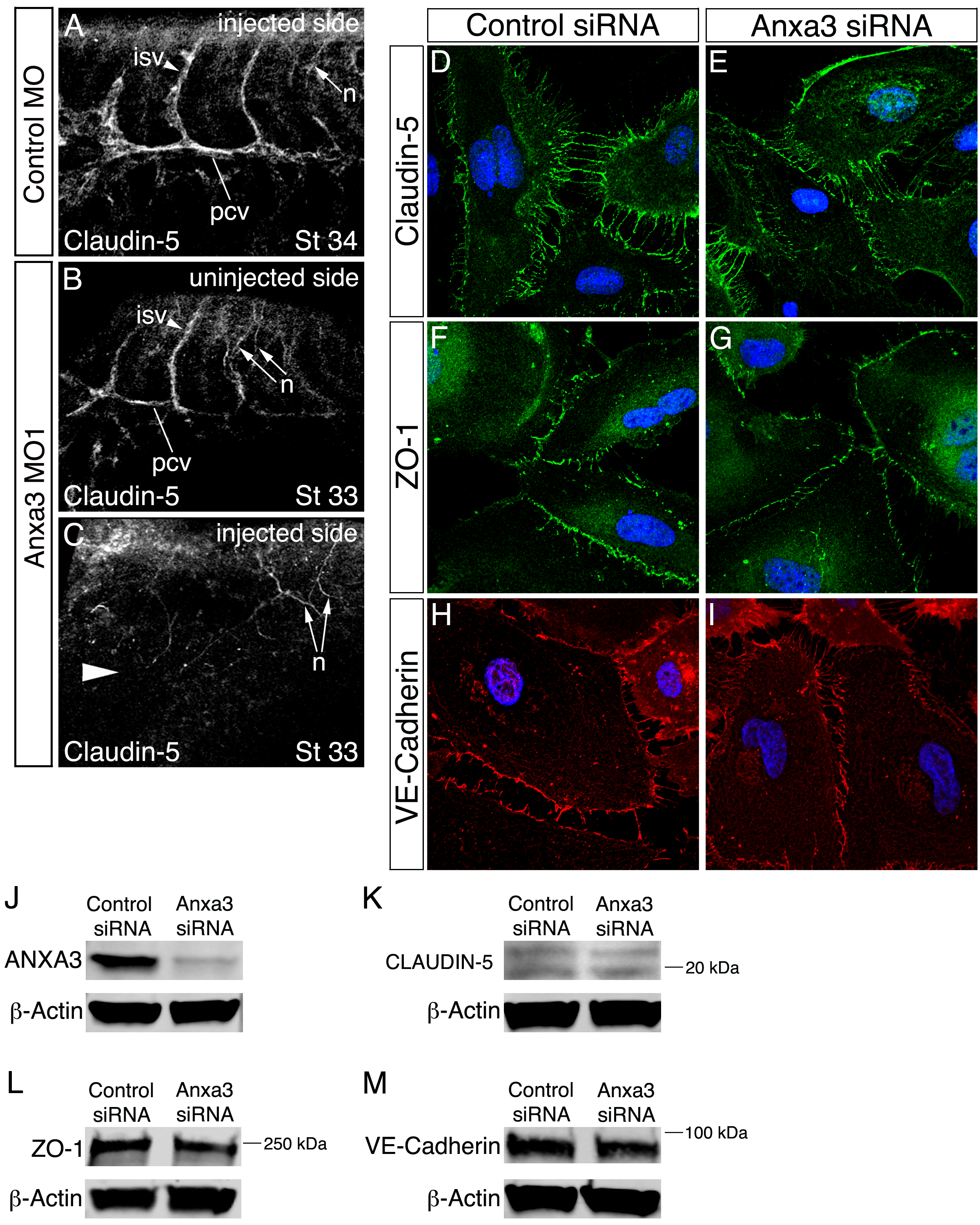

Supplement: S4 Fig — (A) Injected side of a control MO treated embryo (stage 34) immunofluorescently labeled for Claudin-5. Notice expression in the developing posterior cardinal vein (pcv), intersomitic vessels (isv) and neurons (n) extending from the spinal cord. (B) A stage 33, uninjected side of an Anxa3 MO treated embryo, stained for Claudin-5, shows similar levels and localization of Claudin-5 protein compared to A. (C) Immunofluorescent detection of Claudin-5 in the Anxa3 MO injected side of the embryo in B. Note the lack of Claudin-5 in the developing pcv and isv region (large arrowhead), yet expression is present in neurons (n). (D-M) Human umbilical vein endothelial cells (HUVEC) treated with control and Anxa3 siRNAs (35 μM) and analyzed for the tight junction markers, Claudin-5 and ZO1, and the adherens junction marker, VE-Cadherin, via immunofluorescent staining (D-I) and western blot analysis, including ANXA3 (J-M). No detectable changes were observed. Predicted protein sizes: CLAUDIN-5, 23 kDa; ZO-1, 220 kDa; VE-Cadherin, 94 kDa. Note that the CLAUDIN-5 antibody detects two bands at approximately 23 kDa and slightly smaller than 20 kDa). (TIF) [file pone.0132580.s004.tif]

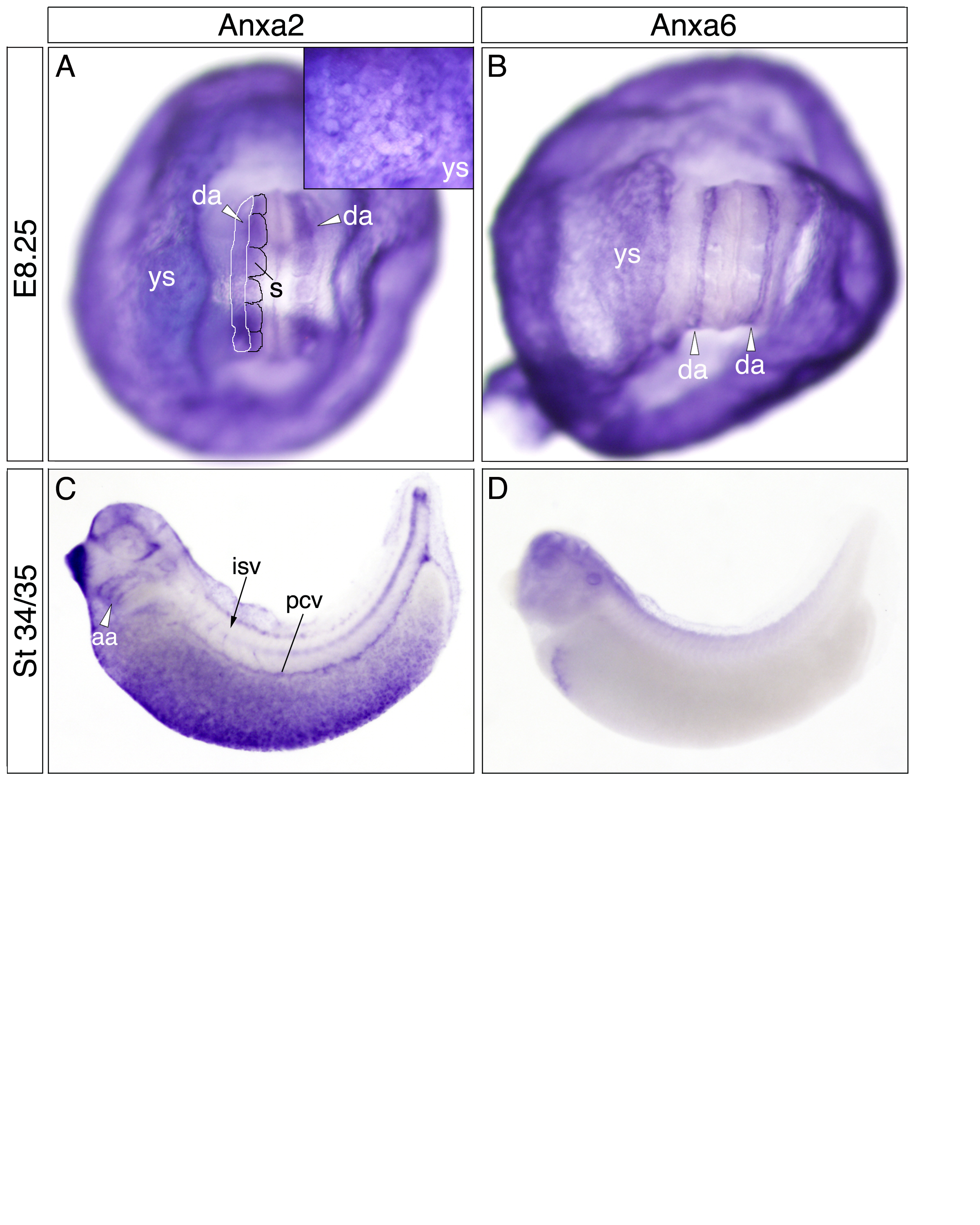

Supplement: S5 Fig — (A-D) Whole-mount in situ hybridization analysis of Anxa2 and Anxa6 transcripts in Xenopus embryos (lateral views) at stages (St) 34–35 and mouse embryos (ventral views) at embryonic (E) day 8.25. (A) Mouse Anxa2 is weakly expressed in the paired dorsal aortae (da, white arrowheads) that lie underneath the Anxa2 expressing somites (s). The da and somites on the right side of the embryo are outlined in white and black, respectively. Anxa2 transcripts can also be detected in the blood vessels of the extra-embryonic yolk sac (ys), inset. (B) Anxa6 RNA is strongly observed in the ys vasculature and the paired da. (C,D) In the frog embryo, Anxa2 transcripts are detected in the intersomitic vessels (isv), posterior cardinal vein (pcv) and aortic arches (aa), while Anxa6 transcripts are not observed in developing blood vessels. (TIF) [file pone.0132580.s005.tif]
